# Nationwide Stature Estimation from Knee Height Measurements in Montenegrin Adolescents

**Published:** 2020-05

**Authors:** Marina VUKOTIC

**Affiliations:** Faculty for Sport and Physical Education, University of Montenegro, Niksic, Montenegro

## Dear Editor-in-Chief

Anthropometry is in various ways significant for the measurement of individual body parts, i.e. for the provision of key data, used to quantitatively determine morphological features and to assess the objective picture of human growth (1). Body height is the main anthropometric parameter for the estimation of individuals and is significant in many situations (2). In addition, it is necessary for the evaluation of child growth for the calculation of nutrition indices of children and adults (3), the prediction and standardization of variables such as lung capacity, muscle strength, the standardization of physical ability measures for the determination of a patient’s proper dose, etc. (4). Furthermore, it can be a good parameter for diagnosing persons with various anomalies and body height loss after doctor medicinal activity on the spine (5), as well as for predicting its loss in the case of the elderly (6).

However, it is not always possible to determine precisely the height of the body, especially including the cases, for example, paralysis, fracture, amputation and various, deformities such as scoliosis, lordosis and kyphosis (7). In such cases, it is necessary to apply some other parameters for the estimation of body height. Variation in relative knee height is a sensitive indicator of early childhood circumstances, but research presents conflicting evidence of how lower leg growth contributes to variability. There is also wide support for the use of relative knee heigh as an indicator of the quality of the environment for growth during infancy, childhood and the juvenile years of development (8).

For this reason, it is very important to establish the relationship between body height and knee heigh in Montenegrins at the national level-not done yet-primarily because in some cases it can be very important to use precisely this anthropometric measure to determine body height, due to the above-mentioned reasons.

Since 2019, the sample in this research comprised 1001 adolescents, all of whom were in their final year of high school (504 males, 497 females) from the territory of Montenegro. The average age of the male subjects was 18.68±0.35 yr; female subjects was 18.70±0.33 yr (age span 18–20). Moreover, it is important to point out that the authors excluded from the study adolescents with body deformities (scoliosis, kyphosis, lordosis, etc.), paralysis, fractures, amputations, and similar.

The study complied with the Declaration of Helsinki. According to Marfell-Jones, Olds, Stewart and Carter (9), anthropometric measurements, including body height and knee heigh, were taken in compliance with the protocol of the International Society for the Advancement of Kinanthropometry (ISAK).

The results correlation coefficients are very high: in the case of male subjects, this coefficient is 0.671, while in the case of female subjects, it amounts to 0.637. The average knee heigh of Montenegrin adolescents equals (males: 47.55±3.08 cm; females: 43.03±2.56 cm), which confirms the main notion of this study that the population of Montenegro has specific body proportions.

Therefore, knee heigh has proven to be a predictor based on which the actual body height can be estimated. The conducted research of knee heigh as a reliable body height predictor is of additional importance, because it is the only research of its kind conducted at the national level in accordance with proportional geographic sampling, which is also of crucial importance for future national and regional research of potential anthropometric predictors.
